# USP38 exacerbates atrial inflammation, fibrosis, and susceptibility to atrial fibrillation after myocardial infarction in mice

**DOI:** 10.1186/s10020-023-00750-2

**Published:** 2023-11-12

**Authors:** Yang Gong, Tingting Yu, Wei Shuai, Tao Chen, Jingjing Zhang, He Huang

**Affiliations:** 1 Department of Cardiology, Renmin Hospital of Wuhan University, 238 Jiefang Road, Wuhan, Hubei 430060 China; 2 Cardiovascular Research Institute of Wuhan University, Wuhan, China; 3 Hubei Key Laboratory of Cardiology, Wuhan, China; 4 Department of Respiratory Medicine, Hubei Veterans Hospital, Wuhan, China

**Keywords:** Atrial fibrillation, Inflammation, Myocardial infarction, Ubiquitin-specific protease

## Abstract

**Background:**

Inflammation plays an important role in the pathogenesis of atrial fibrillation (AF) after myocardial infarction (MI). The role of USP38, a member of the ubiquitin-specific protease family, on MI-induced atrial inflammation, fibrosis, and associated AF is unclear.

**Methods:**

In this study, we surgically constructed a mouse MI model using USP38 cardiac conditional knockout (USP38-CKO) and cardiac-specific overexpression (USP38-TG) mice and applied biochemical, histological, electrophysiological characterization and molecular biology to investigate the effects of USP38 on atrial inflammation, fibrosis, and AF and its mechanisms.

**Results:**

Our results revealed that USP38-CKO attenuates atrial inflammation, thereby ameliorating fibrosis, and abnormal electrophysiologic properties, and reducing susceptibility to AF on day 7 after MI. USP38-TG showed the opposite effect. Mechanistically, The TAK1/NF-κB signaling pathway in the atria was significantly activated after MI, and phosphorylated TAK1, P65, and IκBα protein expression was significantly upregulated. USP38-CKO inhibited the activation of the TAK1/NF-κB signaling pathway, whereas USP38-TG overactivated the TAK1/NF-κB signaling pathway after MI. USP38 is dependent on the TAK1/NF-κB signaling pathway and regulates atrial inflammation, fibrosis, and arrhythmias after MI to some extent.

**Conclusions:**

USP38 plays an important role in atrial inflammation, fibrosis, and AF susceptibility after MI, providing a promising target for the treatment of AF after MI.

**Supplementary Information:**

The online version contains supplementary material available at 10.1186/s10020-023-00750-2.

## Introduction

As the most common arrhythmia, the prevalence of atrial fibrillation (AF) is on the rise worldwide and has become a major public health burden. AF is the most common supraventricular rhythm disturbance following arrhythmic complications of acute coronary syndrome, with an estimated incidence of 6–21%, especially in acute myocardial infarction (MI) (Schmitt et al. 2009). AF is also associated with increased mortality, and in-hospital AF is also associated with increased mortality and in-hospital complications (Berton et al. 2009; Schmitt et al. 2009).

Increasing evidence confirms that cardiac inflammation and secondary interstitial fibrosis are the pathophysiology of early AF in infarction (Hu et al. 2015; Yao et al. 2018). An intense inflammatory response is rapidly triggered after MI, and the early inflammatory response after MI determines the degree of subsequent healing and cardiac remodeling process (Ong et al. 2018). Therefore, early and selective targeting of harmful pro-inflammatory and fibrotic signals is beneficial in reducing the incidence of AF after MI.

The ubiquitin-proteasome system (UPS) is the major non-lysosomal pathway for intracellular protein degradation and plays a major role in the regulation of many cellular processes (Pohl and Dikic 2019). Multiple studies have shown that the ubiquitin-proteasome system plays a role in cardiovascular disease. For example, the E3 ubiquitin ligase WWP2 selectively targets PARP1 for ubiquitination and degradation to regulate isoproterenol-induced cardiac remodeling (Zhang et al. 2020), FAT10 prevents ischemia-induced ventricular arrhythmias by reducing the formation of the Nedd4-2/Nav1.5 complex (Liu et al. 2021), and the muscle-specific ubiquitin ligase atrogin-1 targets signaling proteins involved in cardiac hypertrophy for degradation (Li et al. 2022).

In recent years, the role of ubiquitin-specific protease in cardiovascular disease has received increasing attention as the ubiquitin-proteasome system has been intensively studied. The USP19 attenuates pathological cardiac hypertrophy by inhibiting the TAK1-dependent pathway (Miao et al. 2020), and in addition, Tang et al. found that USP7 reduces myocardial ischemia-reperfusion injury by regulating ferroptosis (Tang et al. 2021), suggesting an important role for the presence of USP in the heart. USP38 is a member of the USP family with cysteine-type endonuclease activity and thiol-dependent ubiquitin acyl hydrolase activity (Chen et al. 2018), and there are limited studies on its function. In recent years, studies have reported that USP38 plays an important role in a variety of diseases. Yi et al. showed that USP38 influences the pulmonary inflammatory response through regulation of IL-33R ubiquitination levels, and that USP38 and KDM5B bind into a unique chromatin-modified complex to suppress excessive inflammatory responses through selective inhibition of pro-inflammatory cytokines (Yi et al. 2022; Zhao et al. 2020), suggesting an important role for USP38 in the regulation of inflammation. It is unclear whether USP38 regulates post-infarction inflammation and thus affects atrial fibrosis and remodeling. Therefore, the aim of this study was to examine the role of USP38 in inflammation and atrial remodeling in MI.

## Materials and methods

### Animals and animal model

All animal handling procedures in this study were in accordance with the Declaration of the National Institutes of Health guide for the care and use of Laboratory animals in Ophthalmic and Vision Research and approved by the Animal Care and Use Committee of Renmin Hospital of Wuhan University under approval number20220301A.

Cardiac-conditional USP38 knockout (USP38-CKO) and USP38 cardiac-specific transgene (USP38-TG) mice were purchased from Cyagen and constructed in a C57BL6/J background by Cre-loxp system technology. The PCR primer for the Cre promoter was: F: 5’-ATGACAGACAGATCCCTCCTATCTCC-3’, R: 5’-CTCATCACTCGTTGCATCATCGAC-3’. The gene mice construction process and schematic diagrams are shown in Supplemental Data S1 and S2. The mice were conditioned in-house and provided with tap water and a commercial diet. Mice were housed at a temperature of 22 ± 2 ◦C and a 12:12-h light/dark cycle with free access to food and water.

MI models were established in adult male mice at 6–8 weeks by ligating the left descending coronary artery (LAD) as previously described (Snider et al. 2021). In brief, mice were anaesthetized with an intraperitoneal injection of 50 mg/kg sodium pentobarbital. After successful anesthesia, artificial respiration was performed to maintain normal pH, PO_2_, and PCO_2_, followed by incision of the pericardium at the third or fourth intercostal space and permanently ligation of the LAD with 7 − 0 silk sutures approximately 2 mm from its origin. The muscle and skin were sutured after the ligation was completed. Electrocardiography (ECG) was recorded synchronously, and changes in the ST-segment were detected. The success of MI construction was judged according to the changes in the ST segment of the ECG and the color of the apex. The sham procedure consisted of a similar operation, except that no ligation was performed. Mice were processed on days 3 and 7 after MI, respectively.

### Echocardiography analysis

Cardiac function was assessed by echocardiography at 7 days after MI. Echocardiography was carried out using the VINNO 6VET ultrasound system (Vinno Technology, Suzhou, Jiangsu, China) which equipped with a 23-MHz linear array transducer (Vinno Technology, Suzhou, Jiangsu, China).

### Cytokine measurement

The blood samples from mice were collected and centrifuged at 3400 rpm (4 °C) for 30 min to obtain serum. Protein concentrations of IL-1β (GEM0002, Servicebio, Wuhan, China), IL-6 (GEM0001, Servicebio, Wuhan, China), and IL-10 (GEM0003, Servicebio, Wuhan, China) in serum were determined using standard ELISA kits according to the instructions.

### Histological analysis

The left atrial (LA) tissue section from day 7 post-MI was embedded in paraffin and cut into 5-µm-thick sections. The degree of LA fibrosis was evaluated using Masson Trichrome staining. Sections were viewed under a microscope and the data were measured with Image Pro-Plus software. Immunofluorescence and histochemical staining analysis was performed to identify macrophages (CD68, iNOS, and CD163), which were immunolabeled with CD68, iNOS, and CD163 antibodies. All images were analyzed by Image-Pro Plus software.

### Electrocardiograph analysis

The ECG telemetry transmitter (China YiSense Biomedical Technology Co., Ltd.) was fixed to the back of the mice, and the leads were placed subcutaneously on the upper right and lower left chest and the ECG was recorded continuously for 24 h. The data were analyzed with LabChart 7 software.

### Electrophysiological studies

Langendorff-perfused hearts were prepared according to our previously published methods (Shuai et al. 2019). In brief, electrophysiological studies in isolated perfused hearts were conducted using the Langendorff apparatus with HEPES-buffered Tyrode’s solution (130 mM NaCl; 5.4 mM KCl; 1.8 mM CaCl2; 1mM MgCl2; 0.3 mM Na2HPO4; 10 mM HEPES; 10 mM glucose; pH adjusted to 7.4 with NaOH), bubbled with 95% O2–5% CO2 at 37 °C and at a constant pressure of 60 mmHg to evaluate the induction of AF, interatrial conduction time (IACT), and atrial effective refractory periods (AERP). Langendorff-perfused hearts were stimulated with a pair of electrodes placed on the right atrial (RA). All isolated hearts were stabilized for 20 min by perfusion at a constant flow before programmed electric stimulation. The hearts that did not recover to a regular spontaneous rhythm or had irreversible myocardial ischemia were discarded.

Teflon-coated (except at the tips) silver bipolar electrodes were placed on the appendages of the RA, LA, and left ventricle. The interelectrode distance between the RA and LA was set at 5 mm to measure the IACT. The ERPs of the left and right atria were measured by the S2 extra-stimulus method using eight regularly paced beats with cycle lengths of 100, 80, 60, and 40 ms. IACT was measured during RA pacing. AF inducibility was tested by burst pacing methods. The induction of AF was tested by applying three chains of a 2-second burst pacing using the electronic stimulator. Specifically, the first 2-second burst had a cycle length (CL) of 40 ms (pulse duration = 5 ms). Following 3 min of stabilization, the second 2-second burst was applied with a CL of 20 ms (pulse duration = 5 ms). After 3 min of stabilization, the last 2-second burst with a CL of 20 ms was applied with a 10 ms pulse duration. AF was defined as a rapid and irregular atrial rhythm with irregular RR intervals lasting at least 1 s. The duration of AF was measured from the end of burst pacing to the first P wave detected after the rapid irregular atrial rhythm.

### Western blot analysis and quantitative real-time PCR

The total proteins were extracted from the frozen LA tissues. Protein concentrations were determined and normalized using the Bicinchoninic Acid (BCA) Protein Assay Kit (G2026, Servicebio, Wuhan, China). Following that, proteins were separated by sodium dodecylsulphate (SDS)-polyacrylamide gel electrophoresis (PAGE), then transferred onto a polyvinylidene difluoride (PVDF) membrane and incubated with primary antibodies (Supplemental Table S1) overnight at 4°C. Finally, secondary antibodies were incubated with the membranes for 60 min at room temperature. Intensive chemiluminescence (BL523B, Biosharp, Anhui, China) was used to visualize the signals. The protein expression levels of target genes were normalized to the internal reference gene GAPDH.

Total RNA was purified from the atrial samples using RNAiso Plus reagent (9109, Takara, Japan). RNA was transcribed into complementary DNA with the PrimeScript RT reagent Kit (#RR047A, TaKaRa). Then, qRT-PCR was conducted in a 20 µl reaction system containing cDNA, forward primers, reverse primers, and SYBR Premix Ex Taq (#RR420A, TaKaRa). The sequences of the primers used for qRT-PCR are described in the Supplemental Table S2.

### Statistical analysis

All the data were expressed as mean ± standard error (SEM) or percentages, and the data were analyzed by GraphPad Prism software (GraphPad Software, San Diego). All data were analyzed by the Shapiro-Wilk normality test for normal distribution. For comparisons between the two groups, a two-tailed Student t-test was applied for normal data, and the Mann-Whitney test was applied for nonnormal data. For comparisons between multiple groups, one-way ANOVA with Bonferroni post hoc analysis was used for normal data, and the Kruskal-Wallis test was used for nonnormal data. Categorical data were analyzed using the Fisher exact test. Values of P < 0.05 were considered to be statistically significant.

## Results

### Alteration of atrial macrophage infiltration and inflammatory response post-MI

**Fig. 1:**
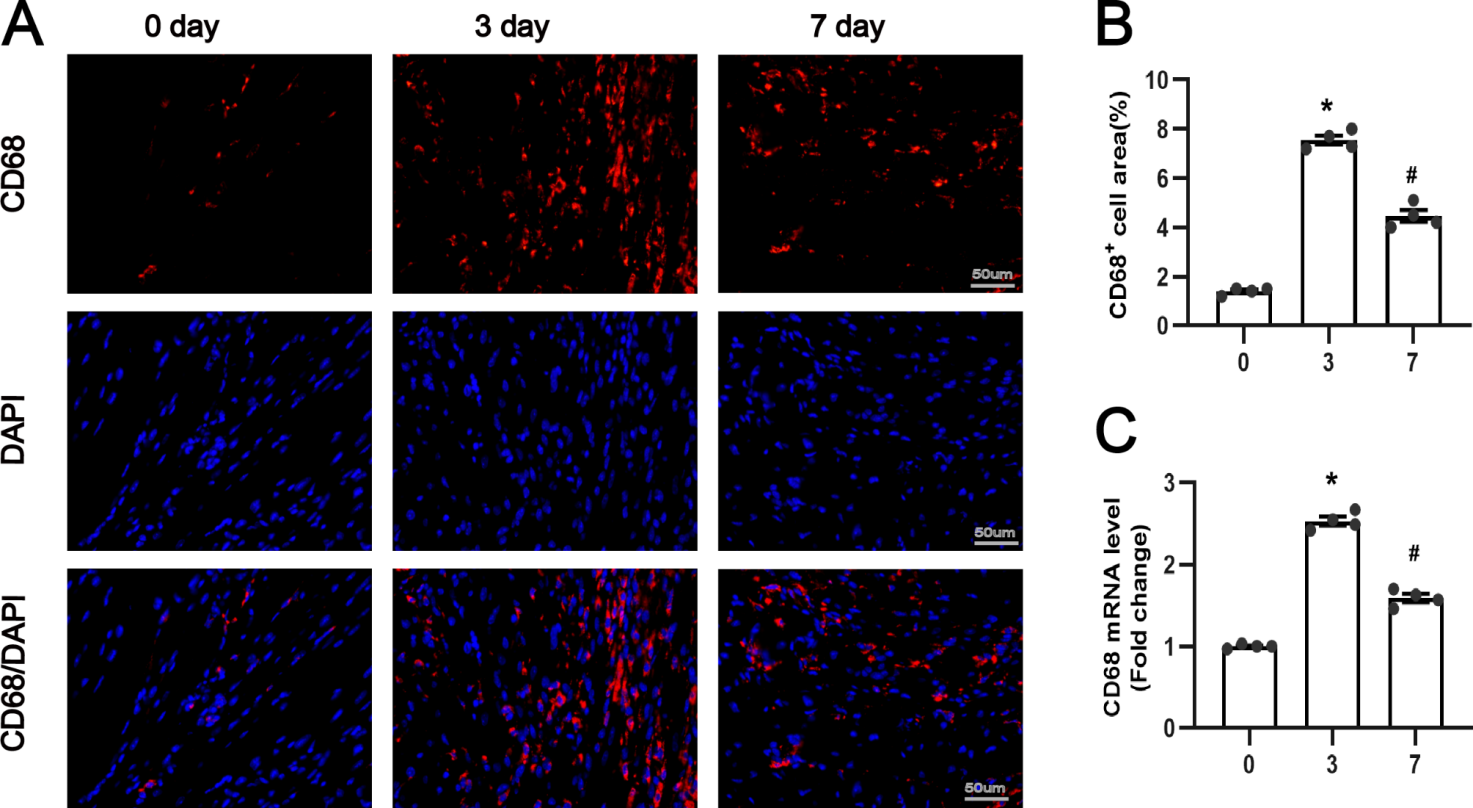
Atrial macrophage infiltration and inflammatory response at 3 or 7 days after MI. (**A**) Representative fluorescent immunostaining images. Left atria at 0, 3, and 7 days after myocardial infarction were stained with anti-CD68 antibody (red) and DAPI (blue). (**B**) quantitative analysis of CD68 + macrophage area. (**C**) quantitative analysis of CD68 mRNA changes by real-time polymerase chain reaction(n = 4). *P < 0.05 vs. 0 day, ^#^P < 0.05 vs.3 day

Due to inflammation plays an important role in the development of AF after MI, we assessed the expression of the M1 macrophage marker CD68 + in the atria at different time periods by immunofluorescence. As shown in Fig. 1A-B, the cell density of CD68 + in the atria of the MI group was significantly increased on days 3 and 7 compared with the sham-operated group, but there were more CD68 + cells on day 3 than on day 7 after MI. In addition, similar results were obtained for the mRNA levels of CD68+ (Fig. 1C). The above results suggest that atrial inflammation is more significant on day 3 than on day 7 after MI.

### USP38 cardiac-conditional knockout attenuates LA inflammation at 3 days post-MI

**Fig. 2:**
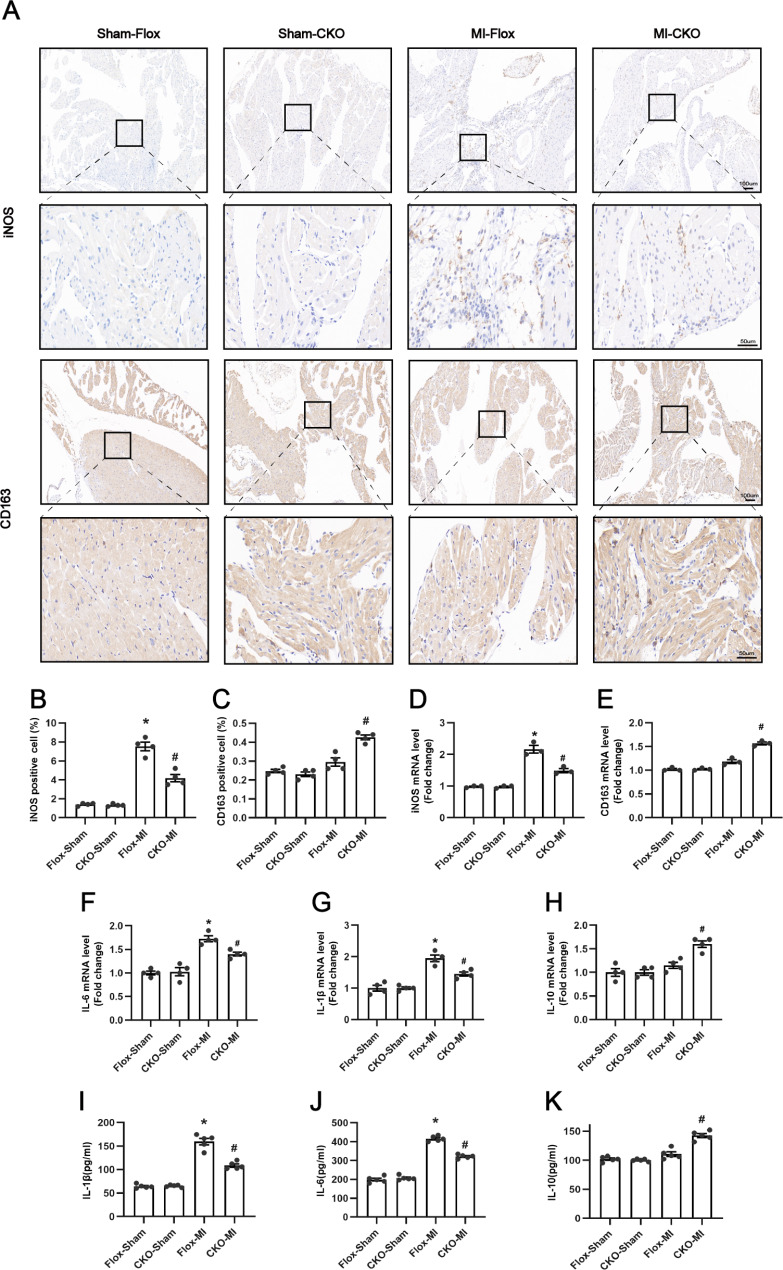
Effect of USP38 cardiac-specific knockout on atrial inflammation after MI. (**A**) Representative immunohistochemical staining images. Left atria 3 days after MI were stained with anti-iNOS and CD163 antibody, respectively. (**B**-**C**) Quantitative analysis of the area of infiltrated iNOS positive and CD163 positive macrophages in the left atrial (n = 4). (**D**-**E**) mRNA expression of iNOS, and CD163 in the left atrial (n = 3). (F-H) mRNA expression levels of IL-1β, IL-6, and IL -10 (n = 4). (I-K) Levels of IL-1β, IL-6, and IL-10 proteins measured by ELISA (n = 5). *P < 0.05 vs. Sham group, ^#^P < 0.05 vs. Flox-MI

M1 macrophages peaked on day 3 after MI and exhibited a pro-inflammatory phenotype. Therefore, we observed the effect of USP38 on atrial inflammation at 3 days post-MI. According to the Immunohistochemistry results, as shown in Fig. 2A, we found that iNOS macrophages were significantly increased in the Flox-MI group of mice compared with the sham-operated group. USP38-CKO significantly decreased iNOS macrophages and increased CD163 macrophages. In addition, the atria in the Flox-MI group showed upregulation of iNOS mRNA expression, whereas USP38-CKO significantly decreased iNOS and increased CD163 mRNA expression (Fig. 2B-E). Also, consistent with the above results, the mRNA expression of IL-1β, IL-6, and IL-10 and the protein levels detected by ELISA showed the same trend (Fig. 2F-K). These results suggest that USP38-CKO can alleviate atrial inflammation on day 3 after infarction.

### USP38 cardiac-conditional knockout improves LA structural remodeling at 7 days post-MI

**Fig. 3:**
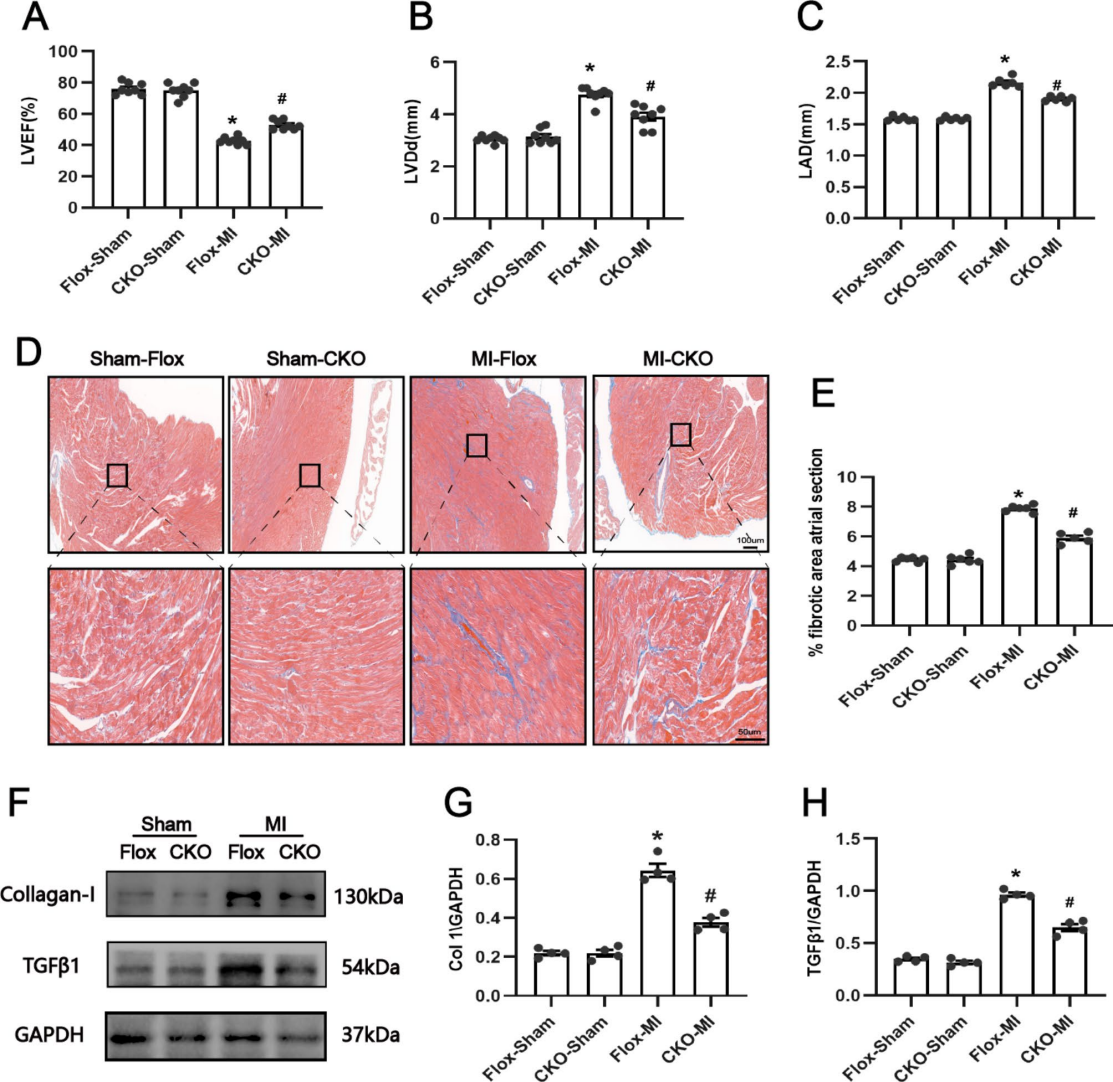
Effect of USP38 cardiac-specific knockout on atrial fibrosis after MI. (**A-C**) Measurement of cardiac function (LVEF, LVDd, and LAD) in mice 7 days after MI (n = 8). (**D**) Representative images of Masson staining of the atrial 7 days after MI. (**E**) Quantitative analysis of atrial fibrosis (%) calculated from Masson staining (n = 6). (**F-H**) Representative Western blotting and statistical analysis of four groups of fibrosis-related proteins (collagen I and TGFβ1) in mice 7 days after MI (n = 4). ^*^P < 0.05 vs. Sham group, ^#^P < 0.05 vs. Flox-MI

To confirm the effect of USP38 on the structural remodeling of the atria after MI, we first performed an analytical assessment by cardiac ultrasound on day 7 after MI. As shown in Figure Fig. 3A-C, mice in the USP38-CKO group exhibited better cardiac function, as well as smaller LA diameter, compared with mice in the Flox-MI group.

We then assessed the extent of atrial fibrosis on day 7 after MI by Masson staining. We observed that the area of LA fibrosis was significantly increased after MI compared with mice in the sham-operated group, whereas USP38-CKO mice exhibited a lower level of atrial fibrosis after MI (P < 0.05) (Fig. 3D-E). In addition, Western blot results showed that the expression of collagen I and TGFβ1 in LA was significantly increased after MI, whereas the expression of collagen I and TGFβ1 in LA was also significantly lower in USP38-CKO mice after MI (Fig. 3F-H). These results suggest that USP38-CKO significantly improved the LA fibrosis after MI.

### USP38 cardiac-conditional knockout improves LA electrical remodeling post-MI at 7 days post-MI

**Fig. 4:**
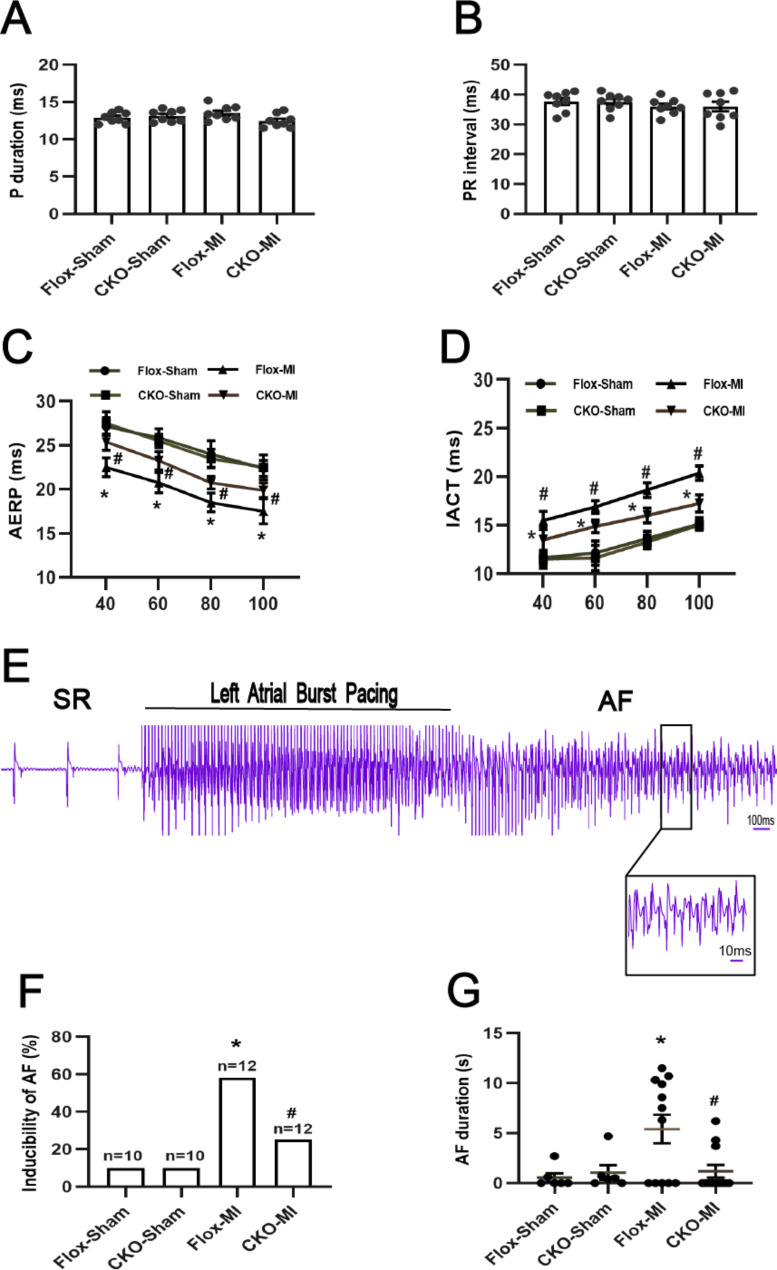
Effect of USP38 cardiac-specific knockout on electrophysiological properties of the atrial in mice after MI. (**A-B**) ECG analysis of P-wave interval and RR interval in each group of mice (n = 8). (**C-D**) IACT and AERP were assessed in the study using isolated perfused hearts of mice (n = 8). (**E**) Representative electrograms (EGMs) of AF induction after atrial burst pacing in CKO-MI mice. (**F-G**) quantitative analysis of AF inducibility and duration in the four groups at 7 days post-MI (n = 10–12). *P < 0.05 vs. Sham group, ^#^P < 0.05 vs. Flox-MI

To clarify the potential influence of USP38-CKO on MI-induced LA electrical remodeling at 7 days after MI. We analyzed the differences in surface ECG between groups of mice. We found no significant differences in the P-wave interval and PR interval between the four groups (Fig. 4A-B). Next, we assessed the alterations in LA electrophysiological characteristics by the Langendorff perfusion system. We found that IACT was significantly prolonged at all basic cycle lengths (basic cycle lengths of 40, 60, 80, and 100 ms) and AERP was significantly shorter after MI compared to the sham-operated group, and USP38-CKO significantly ameliorated these abnormal changes (Fig. 4C-D). In addition, the AF induction rate and duration were significantly increased after MI compared with the sham-operated group. In contrast, the induction rate and duration of AF were significantly lower after MI in USP38-CKO mice (Fig. 4F-G).

Ion channel remodeling after MI has an important role in the development of AF. western blotting showed that the expression of L-type calcium-channel subunit (CaV1.2) in the left atrium was significantly downregulated after MI compared with the sham-operated group, while USP38-CKO significantly upregulated the protein expression of CaV1.2. However, although transient outward potassium channel subunit (Kv4.2 and Kv4.3) expression was reduced after MI, there was no significant difference from the sham-operated group (Supplementary Fig. 4A-D).

### USP38 cardiac-specific overexpression aggravated LA inflammation at 3 days post-MI

**Fig. 5:**
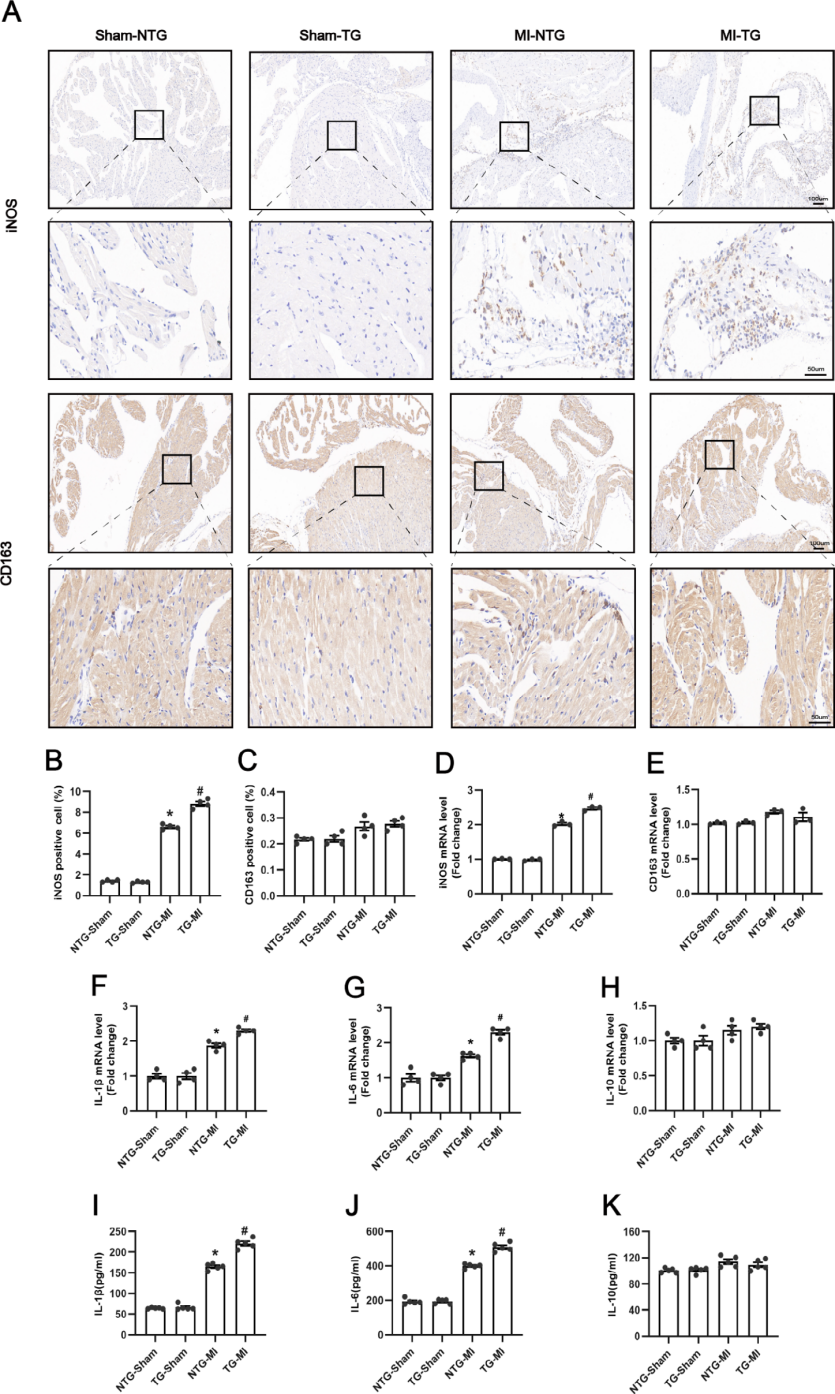
Effect of USP38 cardiac-specific overexpression on atrial inflammation after MI. (**A**) Representative immunohistochemical staining images. Left atria 3 days after MI were stained with anti-iNOS and CD163 antibody, respectively. (**B-C**) Quantitative analysis of the area of infiltrated iNOS positive and CD163 positive macrophages in the left atrial. (n = 4). (**D-E**) mRNA expression of iNOS, and CD163 in the left atrial, respectively (n = 3). (**F-H**) mRNA expression levels of IL-1β, IL-6, and IL -10 (n = 4). (**I-K**) Levels of IL-1β, IL-6, and IL-10 proteins measured by ELISA (n = 5). *P < 0.05 vs. Sham group, ^#^P < 0.05 vs. NTG-MI

The above results suggest that USP38-CKO reduces atrial inflammation after MI; therefore, we explored whether USP38-TG would have the opposite effect. Based on the immunohistochemical results, as shown in Fig. 5A-E, we found that the iNOS macrophages and expression of iNOS mRNA were significantly increased in the NTG-MI group mice compared with the sham-operated group. Overexpression of USP38 significantly increased iNOS expression but had no significant effect on expression with CD163. In addition, the mRNA and protein levels of IL-1β, IL-6, and IL-10 showed the same trend as the immunohistochemical results (Fig. 5F-K).

### USP38 cardiac-specific overexpression aggravated LA remodeling at 7 days post-MI

**Fig. 6:**
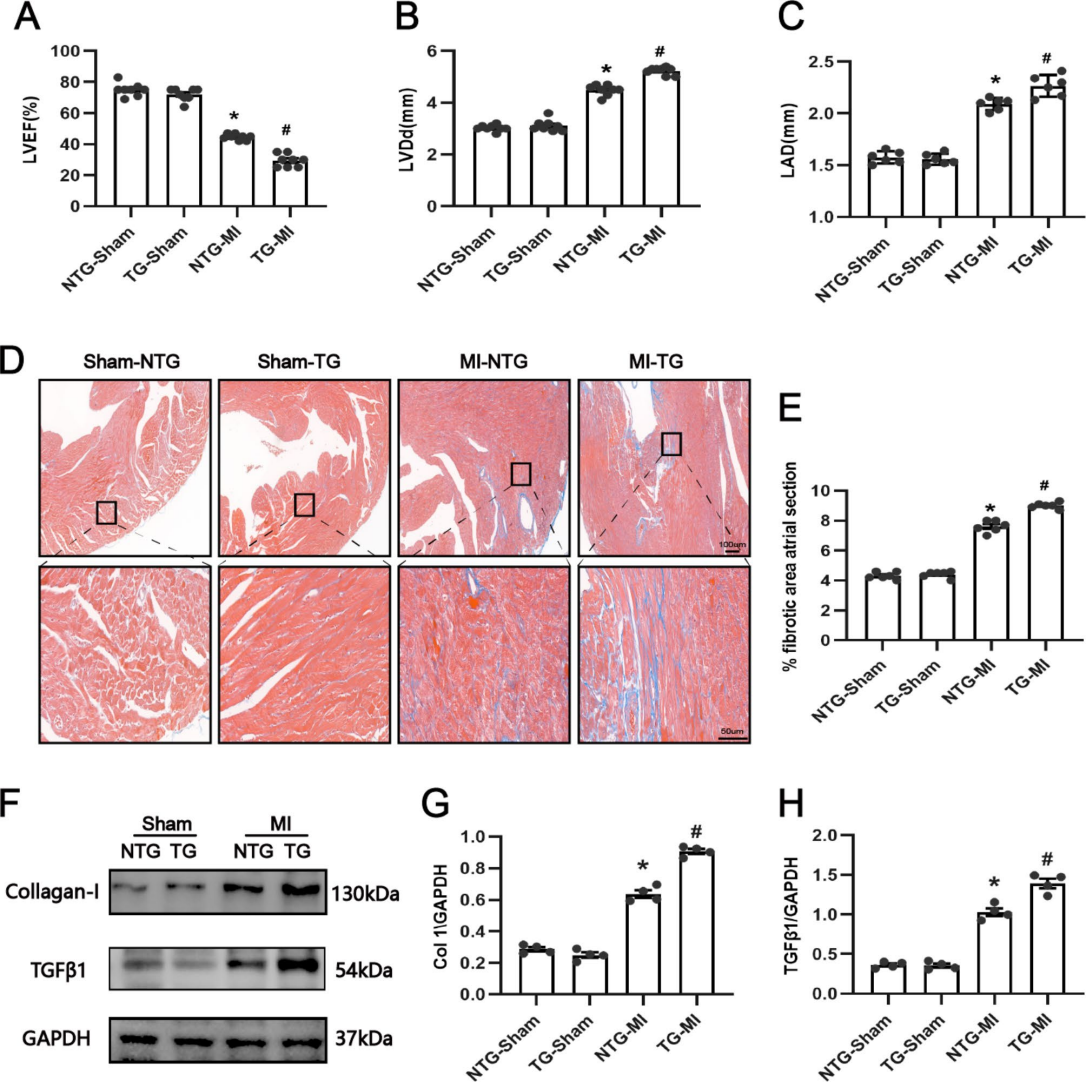
Effect of USP38 cardiac-specific overexpression on atrial fibrosis after MI. (**A-C**) Measurement of cardiac function (LVEF, LVDd, and LAD) in mice 7 days after MI (n = 8). (**D**) Representative images of Masson staining of the atria 7 days after MI. (**E**) Quantitative analysis of atrial fibrosis (%) calculated from Masson staining (n = 6). (**F-H**) Representative Western blotting and statistical analysis of four groups of fibrosis-related proteins (collagen I and TGFβ1) in mice 7 days after MI (n = 4). *P < 0.05 vs. Sham group, ^#^P < 0.05 vs. NTG-MI

Subsequently, we analyzed the effect of USP38-TG on atrial structural remodeling. The cardiac ultrasound results showed no significant difference between cardiac function and LA diameter in the sham-operated group of mice. Compared with mice in the NTG-MI group, TG-MI mice had worse cardiac function and greater LA diameter (Fig. 6A-C). Masson staining suggested that USP38-TG significantly exacerbated the level of atrial fibrosis on day 7 after MI compared with the NTG-MI group (Fig. 6D-E). In addition, the expression of fibrosis indicator proteins showed the same trend (Fig. 6F-H). The results indicated that USP38-TG aggravated the structural remodeling of the atria after MI.

**Fig. 7:**
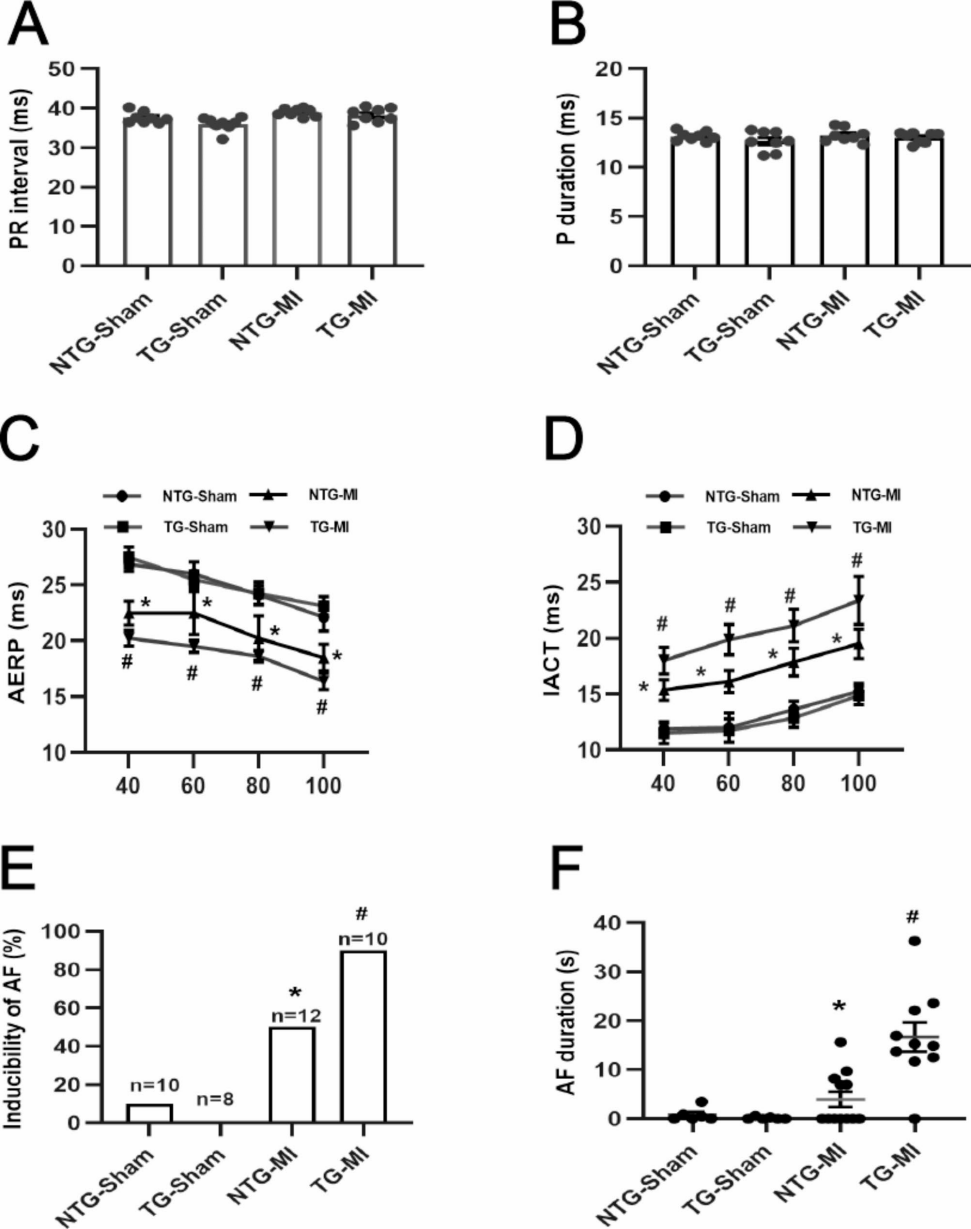
Effect of USP38 cardiac-specific overexpression on electrophysiological properties of the atrial in mice after MI. (**A-B**) ECG analysis of P-wave interval and RR interval in each group of mice (n = 8). (**C-D**) IACT and AERP were assessed in the study using isolated perfused hearts of mice (n = 8). (**E-F**) quantitative analysis of AF inducibility and duration in the four groups at 7 days post-MI (n = 8–12). *P < 0.05 vs. Sham group, ^#^P < 0.05 vs. NTG-MI

Given our findings on the effect of USP38-CKO on atrial electrical remodeling after MI, we next evaluated the effect of USP38-TG on atrial electrical remodeling. We also found no significant differences in the P-wave interval and PR interval between the four groups (Fig. 7A-B). In addition, we found that USP38-TG mice had significantly increased induction rate and duration of AF, prolonged IACT, and significantly shorter AERP compared with NTG-MI group mice (Fig. 7C-F). Also, Western blotting results showed that compared with NTG-MI, USP38-TG significantly downregulated the CaV1.2 protein expression. Similarly, the protein expression levels of Kv4.2 and Kv4.3 were not significantly different in the four groups (Supplementary Fig. 4E-H).

### Effect of USP38 on atrial inflammatory and fibrotic through regulation of TAK1/NF-κB

**Fig. 8:**
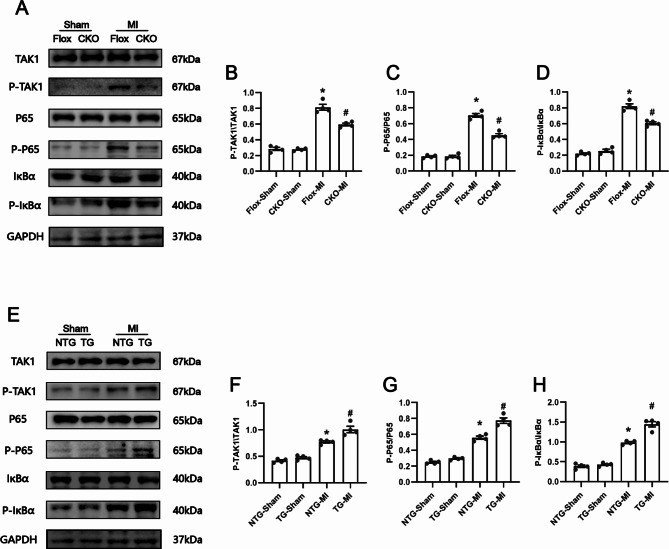
USP38 promotes post-MI inflammation by regulating the activation of the TAK1/NF- k B signaling pathway. (**A**) Representative Western blots of the protein levels (P-TAK1/TAK1, P-P65/P65, P-IκBα/ IκBα) from USP38-CKO mice after MI. (**B-D**) Statistical analysis of the protein levels in the four groups (n = 4). (**E**) Representative Western blots of the protein levels from USP38-TG mice after MI. (**F-H**) Statistical analysis of the protein levels in the four groups (n = 4), *P < 0.05 vs. Sham group, ^#^P < 0.05 vs. Flox-MI or NTG-MI

To assess the molecular mechanisms underlying the potential role of USP38 on atrial inflammation and fibrosis. We evaluated the expression of the TAK1/NF-κB pathway that plays an important role in the inflammatory response. As shown in Fig. 8A-D, Phosphorylated TAK1, NF-κB p65, and IκBα levels were significantly increased in the atria after MI, whereas USP38-CKO significantly decreased the phosphorylated expression levels (Fig. 8A-D). In addition, in USP38-TG mice, we observed results consistent with those described above. The phosphorylation levels of TAK1, NF-κB p65, and IκBα were significantly upregulated in the post-MI atria of USP38-TG group mice compared with NTG-MI group mice (Fig. 8E-H). This data suggests that USP38 regulates atrial inflammation and remodeling by affecting the TAK1/NF-kB pathway to some extent.

## Discussion

In the present study, we explored for the first time the role of USP38 in the pathogenesis of atrial inflammation, fibrosis, and subsequent AF after MI. Our results confirm that knockdown and overexpression of USP38 have no abnormal effects on normal mice. USP38-CKO can improve post-MI cardiac function, fibrosis, and arrhythmogenic substrate of the atria and reduce susceptibility to AF after MI by partially attenuating inflammation, especially macrophage infiltration. In contrast, USP38-TG presents the opposite effect, exacerbating the alterations described above after MI. Mechanistically, USP38 regulates inflammation and atrial remodeling after MI by the TAK1/NF-κB pathway to some extent.

The inflammatory response after MI can promote the development of arrhythmias (Liu et al. 2019; Wei et al. 2021). It is now widely accepted that the atrial inflammatory response leading to AF is associated with structural and electrical remodeling of the atria (Fang et al. 2021; Shuai et al. 2019). In the current study, we also found that higher levels of inflammation in the atria are accompanied by higher levels of fibrosis and that USP38 knockout and overexpression significantly improve and exacerbate LA fibrosis. On the other hand, inflammation can lead to atrial electrical remodeling, including prolongation of IACT and shortening of AERP. Shuai et al. found that atrial inflammation in obese mice led to a significant prolongation of IACT and a significant shortening of AERP (Shuai et al. 2019a), and in the current work, we observed the same phenomenon in the MI model atria. In addition, inflammation can also directly affect the function and expression of ion channels (Scott et al. 2021), where potassium and calcium channels play an important role in AF. In the Ye et al. study, the expression of CaV1.2, a subunit of calcium channels, was significantly reduced in the rat atria after MI (Ye et al. 2019). In the present study, we also observed a significant decrease in CaV1.2 expression in the atrial and the expression of USP38 played a corresponding regulatory role on CaV1.2 expression. In addition, potassium channel protein expression, although reduced, was not statistically different from the sham-operated group. In the study of Beiert et al., the rate and duration of AF induction in mice after MI was also significantly higher, but the mRNA expression of KV4.2 was also not significantly different from the sham group (Beiert et al. 2017), which is consistent with our findings.

USP38, a member of the deubiquitinating enzyme family, has been gaining attraction in recent years. Zhao et al. constructed an endotoxin shock model by intraperitoneal injection of LPS, and USP38-KO mice produced more IL-6 and IL-23α, and their lungs exhibited more severe tissue damage and diffuse inflammation. in addition, they induced acute colitis in mice by dextran sodium sulfate(DSS) model, USP38-KO mice were more susceptible to DSS-induced colitis, with increased colonic inflammation and higher mortality (Zhao et al. 2020). USP38 deficiency promotes downstream pro-inflammatory responses induced by IL-33 in vitro and in vivo. These results suggest that USP38 may act as a regulatory molecule of inflammation (Yi et al. 2022). TAK1, a member of the mitogen-activated protein kinase family, is widely recognized as a key player in pro-inflammatory cytokine signaling and is capable of responding to a variety of stimuli (Lei et al. 2019). Interestingly, NF-κB is a downstream signaling pathway of TAK1, and their binding contributes to a variety of biological processes, such as immune and inflammatory responses. Chen et al. found that Tabersonine reduced the activation of the TAK1/NF-κB signaling pathway and thereby inhibited the cardiac inflammatory response (Chen et al. 2022). In addition, it has been reported that TAK1/ NF-κB is also involved in inflammation. Xu et al. found that high glucose activates macrophages in a TAK1/NF-κB-dependent manner, leading to hyperactivation of inflammation (Xu et al. 2016), and in addition, Hou et al. found that PDG reduced LPS-induced inflammatory responses through downregulation of the AK1-NF-kappaB pathway (Hou et al. 2007; Xu et al. 2022). In the present study, we also observed significant activation of TAK1/NF-κB after MI, accompanied by a significant inflammatory response. USP38 knockout reduced atrial inflammation after MI by downregulating the activation of the TAK1-NF-κB pathway, whereas USP38 overexpression showed the opposite effect. Here we confirmed that USP38 acts as a pro-inflammatory molecule that exacerbates the adverse effects on the atria, but in previous studies, USP38 acted as a negative regulator of inflammation. We speculate that this difference may be a tissue-specific effect. The effect of USP38 on inflammation may be different in different tissues. There are similar reports in previous studies. USP11 deficiency has been reported to have anti-fibrotic and anti-inflammatory effects in a mice model of hyperuricemia nephropathy and folic acid-induced renal fibrosis (Shi et al. 2023).Zhang et al. found that knockdown of USP11 inhibited the release of pro-inflammatory cytokines following cerebral hemorrhage (Zhang et al. 2021). However, recently, it has also been reported that knockdown of USP11 attenuates ischemia-reperfusion-induced cardiomyocyte injury and pro-inflammatory factor secretion (Zhang et al. 2023). The effect of USP11 on inflammation plays a different role in different tissues, which is similar to USP38. In addition, significantly opposite effects of USP25 on the inflammatory response of colon and lung tissues have been reported (Wang et al. 2020; Zhong et al. 2012). Therefore, we suggest that the effect of USP38 on inflammation in the current study differs from previous studies possibly as a tissue-targeting effect. Moreover, differences in experimental modeling may also be responsible.

In the present study, we demonstrated for the first time that USP38, an inflammatory regulatory molecule, can influence atrial inflammation and fibrosis after MI partly through the TAK1/NF-κB signaling pathway. This study suggests that USP38 would be a promising therapeutic target for the upstream prevention of AF after MI.

## Conclusions

We find a novel role for USP38 in regulating post-MI inflammation atrial remodeling through modulation of the TAK1-NF-kB signaling pathway to some extent. These findings provide the first evidence for the involvement of USP38 in the regulation of post-MI AF and strongly suggest that USP38 may provide a therapeutic target for the treatment of post-infarction AF.

### Electronic supplementary material

Below is the link to the electronic supplementary material.

Supplementary Material 1

## Data Availability

Data relevant to this study are available upon reasonable request from the corresponding author.
