# Supplementary material for: USP38 exacerbates atrial inflammation, fibrosis, and susceptibility to atrial fibrillation after myocardial infarction in mice
Source: Mol Med. 2023 Nov 12;29:157. doi: 10.1186/s10020-023-00750-2 (PMC10641944; doi:10.1186/s10020-023-00750-2)
Supplement: Supplementary file 1 — Supplementary Material 1 [file 10020_2023_750_MOESM1_ESM.docx]

**Supplementary materials**

Table S1 Primary antibodies for Western-blots

| primary antibodies | Source organism | producer | Number |
| --- | --- | --- | --- |
| USP38 | Rabbit | Proteintech | 17767-1-AP |
| Collagen I | Rabbit | invitrogen | PA1-26204 |
| TGFβ1 | Rabbit | abcam | ab179695 |
| TAK1 | Rabbit | Proteintech | 12330-2-AP |
| P-TAK1 (Thr 187) | Rabbit | Affinity | AF2019 |
| p65 | Rabbit | CST | #8242S |
| P-p65 | Rabbit | CST | #3031S |
| P-IκBα | Rabbit | CST | #2859S |
| IκBα  CD68  iNOS  CD163 | Rabbit  Mouse  Mouse  Mouse | CST  Servicebio  Servicebio  Servicebio | #4814S  GB113109  GB11119  GB11340 |
| GAPDH | Rabbit | CST | #5174S |

Table S2 Primer sequences for qRT-PCR

| Gene | Forward primer (5’ to 3’) | Reverse primer (5’ to 3’) |
| --- | --- | --- |
| GAPDH | ATCATCCCTGCATCCACT | ATCCACGACGGACACATT |
| CD68 | ACCCGGAGACGACAATCAAC | CTTGGTGGCCTACAGAGTGG |
| CD163 | GGTGGACACAGAATGGTTCTTC | CCAGGAGCGTTAGTGACAGC |
| iNOS | GTTCTCAGCCCAACAATACAAGA | GTGGACGGTGTCGATGTCAC |
| IL-1β | ACAGCAAAAGTTACGGTAGCAG | ATGGGTTCCCCAATGACTTCA |
| IL-6 | CCTGAACCTTCCAAAGATGGC | TTCACCAGGCAAGTCTCCTCA |
| IL-10 | CTTACTGACTGGCATGAGGATCA | GCAGCTCTAGGAGCATGTGG |

**Supplementary Figure 1.** **USP38 cardiac conditional knockout mice construction strategy and schematic diagram**


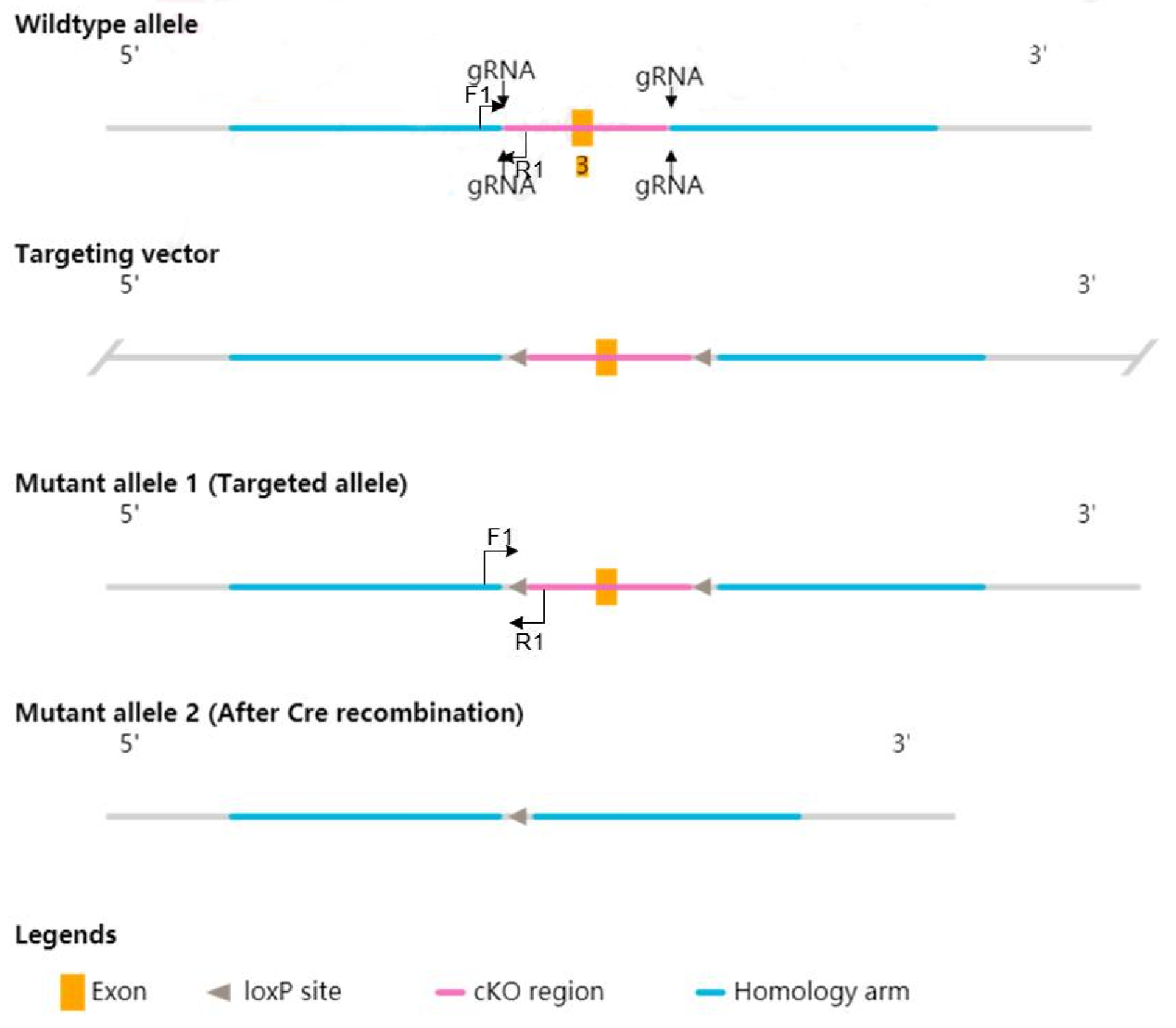


**Supplementary Figure 2.** **USP38 cardiac-specific overexpression mice construction strategy and schematic diagram**


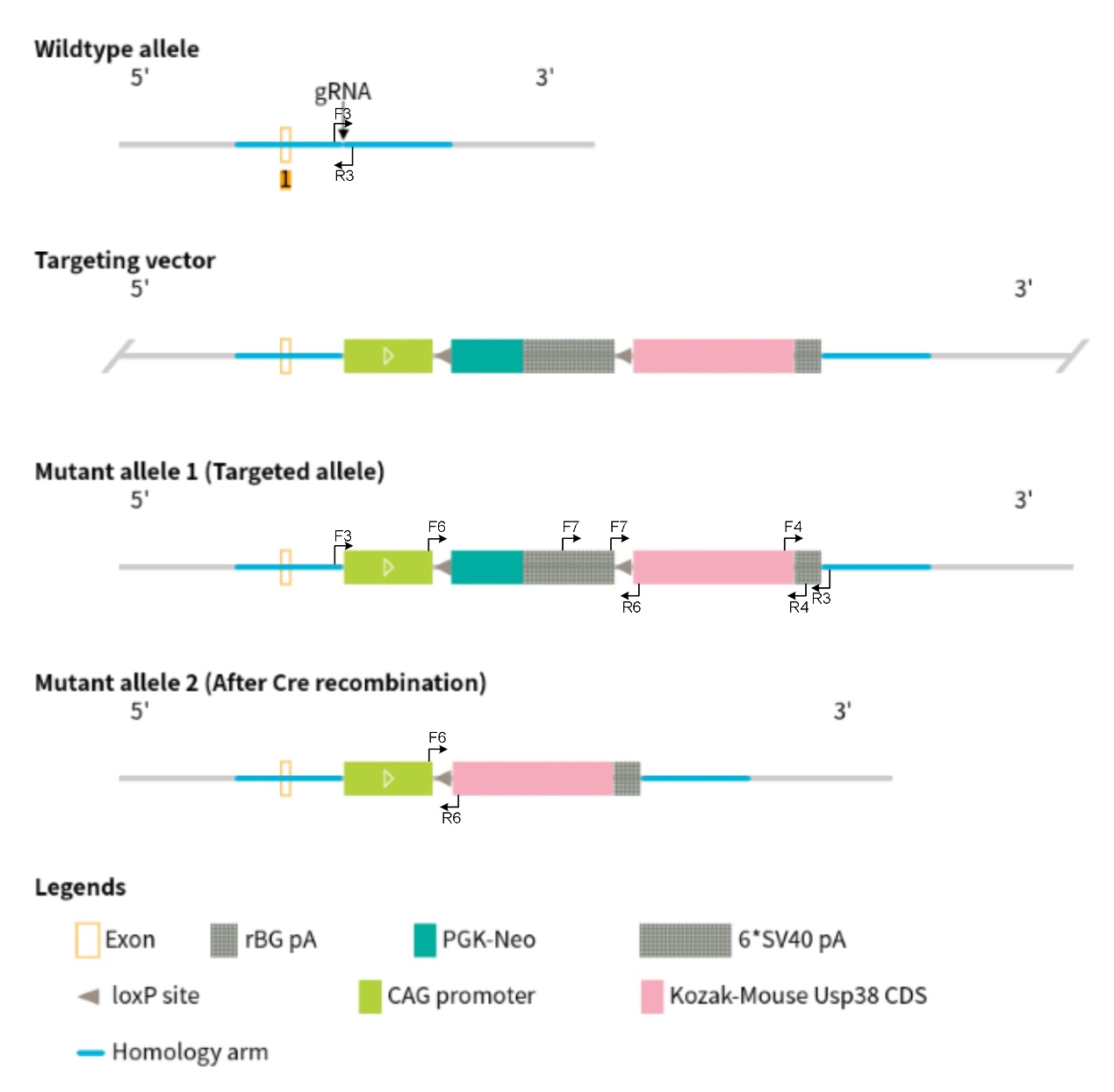


**Supplementary Figure 3.** **Representative western blot images of USP38 in the atria from USP38-CKO (A) and USP38-TG (B) mice**


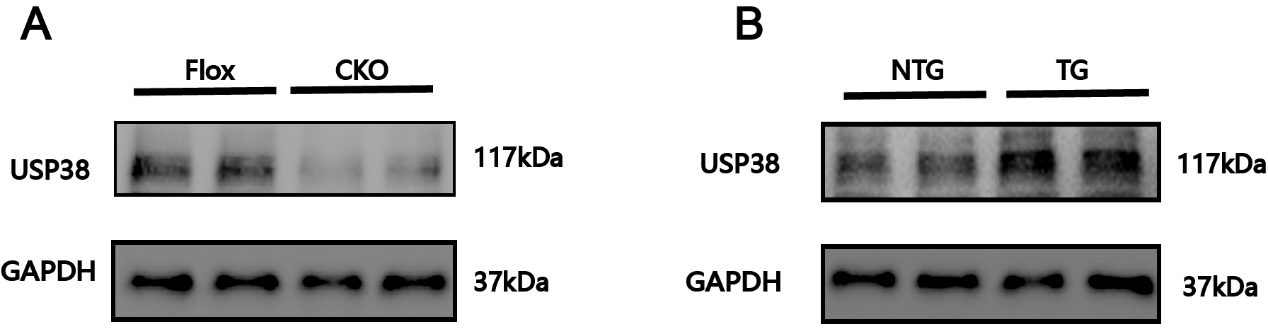


**Supplementary Figure 4**. **Effect of USP38 on atrial ion channel remodeling in mice after MI**


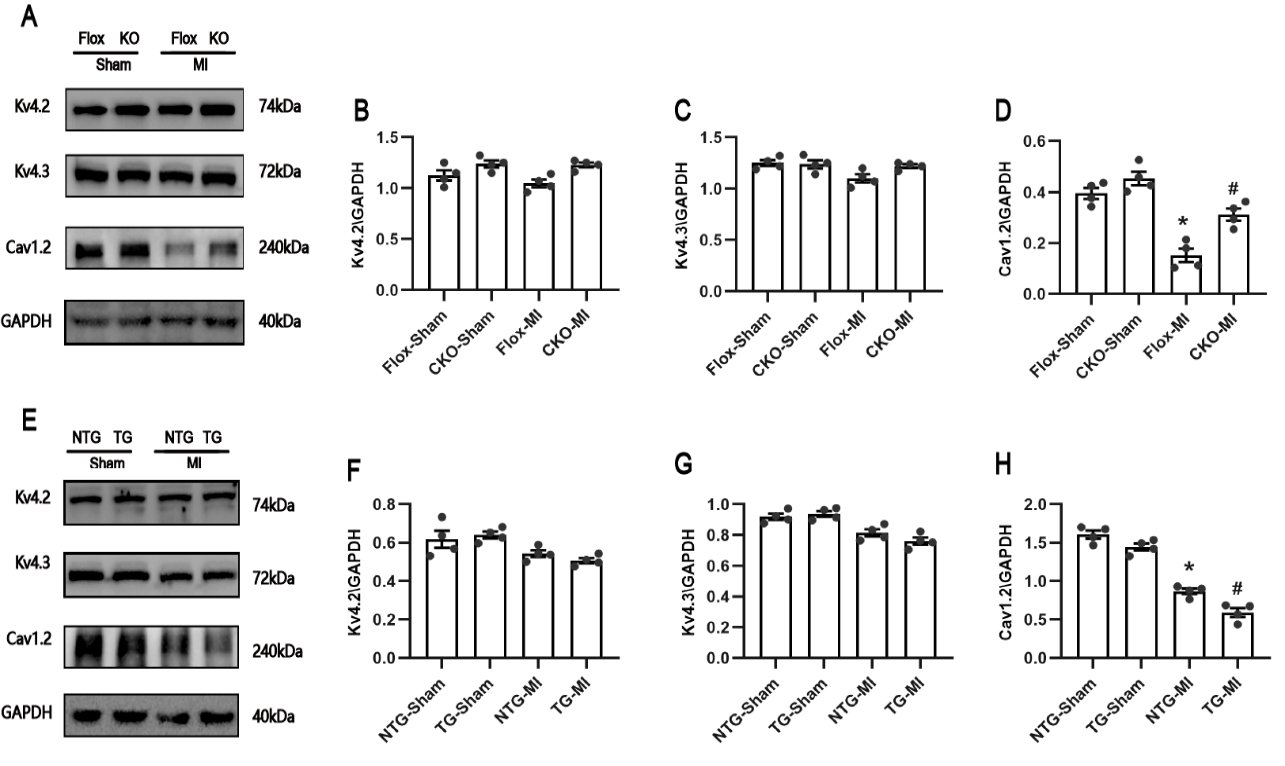


(A and E) Representative Western blots of the ion channel proteins levels (Kv4.2, Kv4.3 and Cav1.2). (B-D) Statistical analysis of USP38-CKO on the ion channel protein expression levels at day 7 after MI (n=4). (F-H) Statistical analysis of USP38-TG on the ion channel protein levels at 7 days after MI (n=4). *P<0.05 vs. Sham group, ^#^P<0.05 vs. Flox-MI or NTG-MI.
